# Efficient Robust Yield Method for Preparing Bacterial Ghosts by *Escherichia coli* Phage ID52 Lysis Protein E

**DOI:** 10.3390/bioengineering9070300

**Published:** 2022-07-07

**Authors:** Yi Ma, Wenjun Zhu, Guanshu Zhu, Yue Xu, Shuyu Li, Rui Chen, Lidan Chen, Jufang Wang

**Affiliations:** 1 School of Biology and Biological Engineering, South China University of Technology, Guangzhou 510006, China; 201920146370@mail.scut.edu.cn (W.Z.); 202021049689@mail.scut.edu.cn (G.Z.); 202121050169@mail.scut.edu.cn (Y.X.); 201936500491@mail.scut.edu.cn (S.L.); 2 Guangdong Provincial Key Laboratory of Fermentation and Enzyme Engineering, South China University of Technology, Guangzhou 510006, China; 3 Bionavi Life Sciences Co., Ltd., Shenzhen 518118, China; chenruicr21@163.com; 4 Department of Laboratory Medicine, General Hospital of Southern Theater Command of PLA, Guangzhou 510010, China; clidangz@163.com

**Keywords:** bacterial ghosts, *E. coli* phage ID52, lysis protein E, lysis activity, L-arabinose

## Abstract

Bacterial ghosts (BGs) are nonliving empty bacterial shells without cytoplasm retaining original morphology and identical antigenicity of natural bacteria, making them high potential and promising vaccine candidates and delivery vehicles. However, the low yield of commonly used BGs preparation methods limits its mass production and widely application. In order to improve BGs production, *E. coli* phage ID52 lysis protein E was introduced to generating BGs for the first time. Above all, we compared the lysis activity of lysis protein of *E. coli* phage φX174 and *E. coli* phage ID52 as well as the effects of promoters on the lysis activity of ID52-E, which shown that the lysis activity and BGs formation rate of protein ID52-E was significantly higher than protein φX174-E. Further, the lysis activity of ID52-E was significantly improved under the control of L-arabinose inducible promoter which initial induction OD_600_ reached as high as 2.0. The applicability of lysis protein ID52-E induced by L-arabinose was proved by preparing probiotic *E. coli* Nissle 1917 BGs and pathogenic *Salmonella typhimurium* BGs in mass production. This paper introduced a novel and highly efficient method for BGs preparation depending on recombinant expression of *E. coli* phage ID52-E under eco-friendly and reasonable price inducer L-arabinose.

## 1. Introduction

Bacterial ghosts (BGs) mainly refer to the inactive hollow cell envelopes derived from Gram-negative bacteria without cytoplasmic contents, which retain the intact cellular morphology and natural surface antigenic structures such as pathogen-associated molecular patterns (PAMPs), which include lipopolysaccharides (LPS), peptidoglycan, monophosphoryl lipid A (MPL), flagella, adhesins and other immune-related stimulating elements of natural bacteria [1]. Therefore, BGs inherit the characteristics of bacterial surface antigenicity and adhesivity [2], making them easy to be recognized and captured by immune cells, and effectively stimulating strong mucosal immunity [3,4], specific humoral and cellular immunity [1,5,6], indicating that BGs can be good candidates for vaccine. And compared with other vaccines that require additional adjuvants [7], BGs have inherent adjuvant components such as LPS and MPL, so BGs don’t need to add additional adjuvants. Since the internal genetic materials were discharged out of the bacteria, BGs don’t have the pathogenicity of natural bacteria, avoiding the problem of virulence reversion, and reducing the risk of virulence gene transfer [8,9]. Furthermore, the special empty cavity structure of BGs has a wide loading capacity, which can load with proteins [10,11], drugs [12,13] and foreign DNA [14]. In addition, antigens can be expressed to the inner membrane, outer membrane and periplasmic space of BGs [15], giving an opportunity to design novel types of polyvalent vaccines and to be an advanced drug delivery system [16]. The simplicity of preparation process and the long shelf life without the need of cold-chain storage on account of the freeze-dry status, make the cost of BGs as vaccines or delivery systems greatly reduced [17].

BGs have so many advantages that they have broad application prospect and great research value, while the low yield of BGs limits the large-scale production and application of BGs. In the short term, BGs were mainly prepared by inducing the expression of *E. coli* phage phiX174 lysis protein gene *E* (*φX174-E*), which under the control of the temperature-inducible expression plasmid pBV220 without adding other chemicals. Contrary to lysis proteins derived from other phages, gene *φX174-E* has no inherent enzymatic function, which encodes a 91-amino acid membrane protein with the competence to fuse the inner and outer membranes of Gram-negative bacteria, giving rise to forming an *E*-specific transmembrane tunnel structure with a size in diameter between 40~400 nm [18] by oligomerizing and co-translationally integrating into the cell membranes [1]. Owing to the difference of osmotic pressure between cytoplasm and surrounding medium, the cytoplasmic contents were expelled to external through transmembrane pores [19]. Scanning electron microscopy (SEM) and transmission electron microscopy (TEM) revealed that the specific transmembrane channels structure of protein E is not randomly distributed on the envelope, but is limited to the potential division sites, mainly in the center or poles of the bacteria [19,20].

However, lysis activity of protein φX174-E depended on the growth phase of bacteria [21]. In a general way, when the optical density value at 600 nm (OD_600_) approximated 0.2~0.6, host bacteria can be lysed effectively by a temperature elevation to 42 °C [22], resulting in a very low yield of BGs, incomplete utilization of medium, and high cost of production. Therefore, if initial induction OD_600_ can be improved during the production of BGs, not only can medium components be fully utilized, but also can the yield of BGs be significantly increased. In order to improve the yield of BGs, Yu et al. [23] obtained a mutant mE of lysis gene φX174-E using gene recombination technology, whose initial induced lysis OD_600_ was up to 1.1. Further, Zhu et al. [22] constructed a fusion expression vector containing the fusion gene of mutant gene mE and *Staphylococcus aureus* nuclease A gene, which could effectively induce host bacteria to lysis with the intracellular degradation of genetic material of host cells, and initial induced lysis OD_600_ could reach as high as 1.2.

However, the BGs yield of above preparation methods was still far from large scale production. Based on this, Ma et al. [24] screened a mutant protein E_M_ by site-directed mutation of φX174-E, and the initial induced OD_600_ could reach 2.0 with the joint control of T7 promoter and pLysS plasmid, achieving efficient preparation of *E. coli* and *Salmonella enteritidis*. However, the inducer IPTG had a certain toxic effect, and the high price and cost of IPTG were not conducive to the large-scale production and application of BGs. Furthermore, Ma et al. [24] continued to mutate on the basis of the mutant protein E_M_ without screening the mutant protein with high lysis activity. Therefore, it can be seen that the possibility of improving the yield of BGs by mutating lysis gene *φX174-E* was low, and this method has been in a bottleneck period. Some chemical agents such as NaOH, H_2_O_2_ and SDS have been proved to be an alternative method to commonly method involving protein E for producing BGs [25]. But the antigenic components LPS was destroyed after treated with NaOH, which may reduce the immunogenicity [26]. Therefore, there is still a long way to go to find a safer, effective and low-cost method for the preparation of BGs.

The *E. coli* Nissle 1917 (EcN), a widely used probiotic, not only possesses the special lipopolysaccharide (LPS) in outer membrane, which exhibits a good immunomodulating property, but also can colonize and replicate in tumors efficiently [27]. Therefore, EcN ghosts can be excellent drug delivery vehicles in targeting therapy of tumor. *Salmonella typhimurium* (ST), an important food-borne non-host-adapted Gram-negative intracellular pathogen, has competence to infect a variety of animal hosts and humans around the world. Vaccination is one of the effective methods to reduce the infection of ST in animals [28], and it also reduces the risk of human infection from food infected with ST. Studies have shown that ST ghosts can effectively protect animals from infection by ST [29]. However, there is no effective method to produce EcN ghosts and ST ghosts with high yield. Therefore, it is of great significance to improve the yield of EcN ghosts and ST ghosts to facilitate the large-scale application of BGs.

Like *E. coli* phage phiX174, *E. coli* phage ID52 also belongs to the *Microvirus* family, and it also contains lysis protein gene *gpE* (*ID52-E*), which encodes a 103-amino acid [30]. According to the protein homology comparison, the homology of lysis protein ID52-E and φX174-E was 55.34%, so protein ID52-E also has the potential to forming BGs. However, there are few reports on the lysis activity of protein ID52-E. Thus, this paper focuses on the lysis effect of *E. coli* phage ID52 lysis protein ID52-E to detect whether it can be applied to produce BGs and improve the yield of BGs. First, the lysis gene *φX174-E* and lysis gene *ID52-E* were cloned into pBV220-Chl plasmid to compare the lysis effects of two lysis proteins, whose results showed that the lysis effect of lysis protein ID52-E was significantly higher than that of φX174-E. In order to test whether the yield of BGs could be further improved, the temperature inducible promoter of pBV220 was replaced with L-arabinose inducible promoter and the results manifested that the OD_600_ initiation can reach as high as 2.0, while the minimum OD_600_ can be reduced to 0.3~0.4 after lysis. Finally, in order to test the feasibility of the efficient BGs preparation system by protein ID52-E under control of L-arabinose inducible promoter, the system was applied to prepare EcN ghosts and ST ghosts, respectively. And the results manifested that protein ID52-E could be applied to the high-efficiency and high-yield production of EcN ghosts And ST ghosts, whose initial induced OD_600_ could be as high as 2.5.

## 2. Materials and Methods

### 2.1. Bacterial Strains, Plasmids, and Culture Conditions

The Bacterial strains, plasmids, and primers used in this research were listed in Table 1. LB (Luria-Bertani) medium used in this research was purchased from Beijing Land Bridge (Beijing, China) for bacterial growth. Bacteria containing pBV220 plasmids were cultured at 30 °C and 220 rpm, and protein expression was induced at 42 °C and 220 rpm. All other bacteria were cultured at 37 °C and 220 rpm unless otherwise indicated. Furthermore, L-arabinose, chloramphenicol (Chl^+^), and other reagents used in this study were purchased from Sangon Biotech (Shanghai, China) unless otherwise specified. The final concentrations of chloramphenicol, kanamycin and ampicillin in LB were 25 μg/mL (Chl^+^), 50 μg/mL (Kan^+^) and 100 μg/mL (Amp^+^), respectively.

### 2.2. Lysis Protein E Expressing Plasmid Construction

Plasmid pBV220-φX174-E and pBV220-ID52-E were constructed by restriction-free cloning technology (RF cloning) [31,32] (Figure S1A,B in Supplementary Materials). First, using plasmid pET29a-φX174-E and pET29a-ID52-E as template, lysis gene *φX174-E* and lysis gene *ID52-E* were amplified by PCR with primer pairs of φX174-E-F/φ174-E-R and ID52-E-F/ID52-E-R, respectively. Then PCR products were detected by 1% agarose gel and purified and recovered by DNA Gel Extraction Kit (TIANGEN BIOTECH, Beijing, China). Next, using the purified lysis gene *φX174-E* and the purified lysis gene *ID52-E* fragment as a pair of primers in a linear amplification reaction around a circular plasmid pBV220-sGFP-Chl, respectively. Later, the methylated parental plasmid was digested by treating the linear amplification reaction products with *Dpn* I enzyme. Then the reaction mixture was transformed into *E. coli* DH5α competent cells. Positive clones were confirmed by DNA sequencing (TIANYI HUIYUAN, Guangzhou, China). The resulting plasmids were designated as pBV220-φX174-E and pBV220-ID52-E.

Plasmid araC-ParaBAD-ID52-E was constructed by seamless assembly cloning with Seamless Assembly Cloning kit (Taihe Biotechnology, Beijing, China) (Figure S1C in Supplementary Materials). Firstly, using the plasmid pBV220-ID52-E as template, a pair of primer linearized pBV220-ID52-E-F/linearized pBV220-ID52-E-R was used for amplifying linearized fragment of pBV220-ID52-E, whose temperature-sensitive lambda-repressor CI857 gene was deleted. Next, the L-arabinose inducible promoter *araC-ParaBAD* was amplified from the plasmid pKD46 using primers “araC-ParaBAD-F/araC-ParaBAD-R”. Finally, seamless Assembly Cloning kit was used for seamless assembly cloning of linearized fragment pBV220-ID52-E and L-arabinose inducible promoter *araC-ParaBAD*. Then the reaction mixture was transformed into *E. coli* DH5α competent cells. Positive clones were confirmed by DNA sequencing (TIANYI HUIYUAN, Guangzhou, China). The resulting plasmids were designated as araC-ParaBAD-ID52-E. Primers required to the above vectors were listed in Table 1.

### 2.3. Induction Expression of Lysis Protein E

The three aforementioned plasmids were transformed into *E. coli* BL21(DE3) competent cells, respectively. Next, the recombinant strains containing different plasmid were cultured to different OD_600_ values, and the expression of lysis proteins in bacteria containing plasmid pBV220-φX174-E or pBV220-ID52-E was activated by shift the temperature to 42 °C, while L-arabinose with final concentration of 0.5 mg/mL was added to activate the expression of lysis protein ID52-E in bacteria containing plasmid araC-ParaBAD-ID52-E.

On the one hand, the decrease degree of OD_600_ value is one of the indicators to evaluate the lysis activity of different lysis proteins E in *E. coli* BL21(DE3). The values of OD_600_ were detected at initial induction OD_600_ approximately 0.8, 1.2, 1.6, 2.0 and detected every 30 min after inducing the expression of lysis protein E. And the OD_600_ decrease degree was calculated by the following formula: OD_600_ decrease degree % = (1 − post-lysis OD_600_/pre-lysis OD_600_) × 100%. On the other hand, the lysis efficiency of different lysis proteins E was evaluated by detecting viable cell counts before and after lysis, which was conducted by serial dilution and plate count methods. And the lysis efficiency was calculated by the following formula: lysis efficiency % = (1 − post-lysis CFU/pre-lysis CFU) × 100%.

Furthermore, the outflow of genome and intracellular protein after lysis were another indicator to evaluate the lysis activity of lysis protein. In order to detect the outflow of the genome after lysis, *E. coli* BL21(DE3) harboring pBV220-φX174-E, pBV220-ID52-E and araC-ParaBAD-ID52-E plasmid respectively was cultured to OD_600_ approximately 1.6 to induce the expression of lysis protein. Then 2 mL bacterial liquid before and after lysis were collected respectively and centrifuged at 5000 rpm for 20 min to collect the bacterial deposits whose genome were extracted by Rapid Bacterial Genomic DNA Isolation Kit (Sangon Biotech, Guangzhou, China) and the genome concentration was measured. The degree of genome leakage was calculated by the following formula: genome leakage degree % = (1 − post-lysis genome concentration/pre-lysis genome concentration) × 100%. All experiments were conducted with three biological replicates.

At the same time, the protein leakage after lysis was detected by SDS-PAGE. *E. coli* BL21(DE3) harboring pBV220-φX174-E, pBV220-ID52-E and araC-ParaBAD-ID52-E plasmid respectively was cultured to OD_600_ approximately 1.6 to induce the expression of lysis protein. Then 5 mL bacterial liquid before and after lysis were collected respectively and centrifuged at 5000 rpm for 20 min to collect the bacterial deposits and culture supernatant. The bacterial deposits were re-suspended with 500 μL PBS and ultrasonic crushing was performed. Then the bacterial deposits sample after crushing and the culture supernatant sample were detected by SDS-PAGE.

### 2.4. Scanning Electron Microscopy (SEM)

*E. coli* BL21(DE3) harboring different plasmids respectively was cultured to OD_600_ approximately 1.6 to induce the expression of lysis protein Then 10 mL bacterial liquid was collected before lysis and after lysis and the bacteria cells were pelleted by centrifuging at 5000 rpm for 20 min at 4 °C, respectively. Then the precipitate was washed three times with sterile phosphate-buffered saline (PBS) to fully remove the medium. Next, the pellets were resuspended in 5 mL 2.5% glutaraldehyde special electron microscopic fixative and fixed at 4 °C for 8 h. After fixation, cells were washed 3 times with deionized water and eventually resuspended in 1 mL deionized water. Drop samples to the dried cell strip and embed them in filter paper. Subsequently, samples were dehydrated with 70%, 85% and 95% ethanol step by step for 15 min each time, and soaked with 100% ethanol for 15 min (repeat this step 3 times). Next, samples were dried with critical point dryer named Autosamdri-815 and following were coated by a gold sputter coater. Finally, the SEM images were acquired using Merlin SEM (Zeiss, Oberkochen, Germany).

### 2.5. Transmission Electron Microscopy (TEM)

*E. coli* BL21(DE3) harboring different plasmids respectively was cultured to OD_600_ approximately 1.6 to induce the expression of lysis protein Then 10 mL bacterial liquid was collected before lysis and after lysis and the bacteria cells were pelleted by centrifuging at 5000 rpm for 20 min at 4 °C, respectively. Same as SEM sample pretreatment, the pellets were washed three times with sterile PBS and fixed with 5 mL 2.5% glutaraldehyde special electron microscopic fixative at 4 °C for 8 h. Then samples were washed three times with deionized water to get rid of fixative. Eventually, 1 mL sterile ddH_2_O was used to resuspend the sample. Placed a clean piece of filter paper in the culture dish and placed the carbon coated copper mesh (face up) on top of the filter paper. Added 20 µL bacterial droplets to the carbon film copper mesh for 5~10 min at room temperature. Gently moved the copper mesh with tweezers so that the excess bacteria droplets on the copper net were absorbed by the filter paper. Then dropped 20 µL of 3% phosphotungstic acid staining solution to the carbon coated copper mesh with bacteria. Finally, the samples were visualized under a transmission electron microscope (ThermoFisher scientific, Talos L120C, Waltham, MA, USA).

### 2.6. Western Blot Analysis

Western blot analysis in this research was performed according to an established procedure with slight modifications [24]. In brief, the samples at different induction time points was harvested by centrifuging at 5000 rpm for 15 min. Then the pellets were resuspended in PBS and the precipitates were ultrasonic broken for 100 W, 5 min. The crushed bacterial liquid was centrifuged at 17,000× *g* for 90 min at 4 °C to obtain supernatant and precipitation. Added 5× loading buffer to the whole liquid, supernatant and precipitation in proportion, and boiled for 10 min at 100 °C, respectively. 10 µL protein samples of *E. coli* BL21(DE3) was separated by 16% tricine-SDS-PAGE. After electrophoresis, proteins bands on the gel were blotted onto a nitrocellulose (NC) membrane (0.2 µm, GE Healthcare Bio-Sciences, Pittsburgh, PA, USA) using the Bio-Rad Transblot Turbo system at 80 V for 1.5 h. Following washed with TBST buffer (Sangon Biotech, Shanghai, China) for 10 min, the NC membrane was incubated with blocking buffer (5% (*w*/*v*) skimmed milk powder in TBST) for 2 h to block the reactive sites of the NC membrane. Then the NC membrane was washed with TBST for three times to get rid of superfluous milk, 10 min each time. Later, the NC membrane was incubated with Anti-Strep-Tag II Monoclonal Antibody 8C12 produced in mouse (AmyJet Scientific, Wuhan, China) at 1:5000 in TBST at 4 °C overnight. The following day, the NC membrane was washed with TBST for three times to remove surplus primary antibody, 10 min each time. The NC membrane was incubated subsequently with horseradish peroxidase (HRP)-conjugated Goat Anti-Mouse IgG H&L (Abcam, Cambridge, MA, USA) at 1:3000 in TBST at room temperature for 2 h. Following repeated washing with TBST, the Super Signal West Pico Chemiluminescent Substrate (ThermoFisher scientific, Waltham, MA, USA) was used to visualize the labeled bands on the NC membrane.

### 2.7. Construction of EcN with araBAD Deletion Mutation and ST with araBAD Deletion Mutation

*E. coli* Nissle 1917 and *Salmonella Typhimurium* engineering strain with *araBAD* gene deletion were constructed by using λ Red homologous recombination system. Firstly, the pKD46 plasmid was transformed into EcN or ST competent cells by electroporation with 2.0 kV and the recombinant positive clones were screened by ampicillin and colony PCR by P1/P2. Then the FRT-kanamycin-FRT linear fragment was amplified by using P3/P4 (EcN) and P5/P6 (ST) containing 56-nt homology arms targeting the *araBAD* gene as primers and pKD4 plasmid as PCR template following purified by DNA Gel Extraction Kit (Sangon Biotech, Guangzhou, China). The EcN or ST recombination clones containing pKD46 plasmid was inoculated into LB liquid medium with 100 µg/mL Amp^+^ at 30 °C. The next day, the cells were transferred to fresh LB broth and cultured at 30 °C and 220 rpm until OD reached 0.3~0.4, then L-arabinose with final concentration of 0.5 mg/mL was added to continue culturing until OD reached 0.5~0.6. Subsequently, the cells were made into electrocompetent cells and were transformed with 500 ng purified FRT-kanamycin-FRT linear fragment. Colony PCR was performed to verify whether *araBAD* gene was successfully replaced by kanamycin fragment by primers P7/P8 (EcN) and P9/P10 (ST) on the colonies grown on ampicillin and kanamycin double resistant plates, respectively. Nextly, the strain EcN*ΔaraBAD::kanamycin* and the strain ST*ΔaraBAD::kanamycin* were obtained by culturing at LB broth at 42 °C for 12 h to remove the pKD46 plasmid. Then the plasmid pCP20 was transformed into the strain EcN*ΔaraBAD::kanamycin* and ST*ΔaraBAD::*kanamycin competent cells by electroporation with 2.0 kV, respectively, which could express FLP recombinase at 42 °C to remove kanamycin resistance genes inserted into the genome and disappeared with the cell division and proliferation. The successful transfer of pCP20 plasmid was achieved by colony PCR using P11/P12 as primers. Finally, the EcN engineering strain and ST engineering strain with ara*BAD* gene deletion named EcN*ΔaraBAD::FRT* and ST*ΔaraBAD::FRT* were identified by PCR using primers P7/P8 and P9/P10 according to the nucleotide sequence of EcN and ST available in NCBI (EcN: NZ_CP007799.1; ST: NZ_CP034230.1). The lysis curves of EcN*ΔaraBAD:FRT* engineering bacteria and ST*ΔaraBAD::FRT* engineering bacteria were plotted by measuring OD_600_. The morphological observation and identification of EcN*ΔaraBAD:FRT* BGs and ST*ΔaraBAD::FRT* BGs were performed by SEM and TEM.

### 2.8. Statistical Analysis

All statistical analyses were performed with GraphPad Prism 8.0.2 software and OriginPro 2021. The Data were expressed in the format of mean ± S.D, and the significance level was analyzed by ordinary one-way ANOVA with Tukey multiple comparison.

## 3. Results

### 3.1. Lysis Protein E Expressing Plasmid Construction

The specific primers were used to conduct colony PCR on the positive clones obtained from the transformation products to verify whether the construction of lysis protein E expressing plasmid was successful. Bands of approximately 343 bp and bands of approximately 376 bp were visualized in positive clones harboring pBV220-φX174-E and pBV220-ID52-E by PCR using φX174-E-F/φX174-E-F and ID52-E-F/ID52-E-R primers, respectively (Figure S2A in Supplementary Materials). Bands of approximately 1631 bp were visualized in positive clones containing araC-ParaBAD-ID52-E by PCR using araC-ParaBAD-F and ID52-E-R primers (Figure S2B in Supplementary Materials).

### 3.2. Lysis Activity of Protein φ174-E and ID52-E in E. coli BL21(DE3)

**Figure 1:**
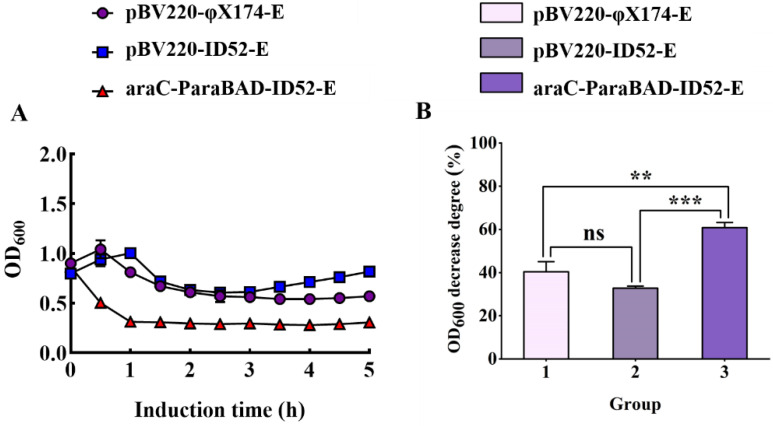

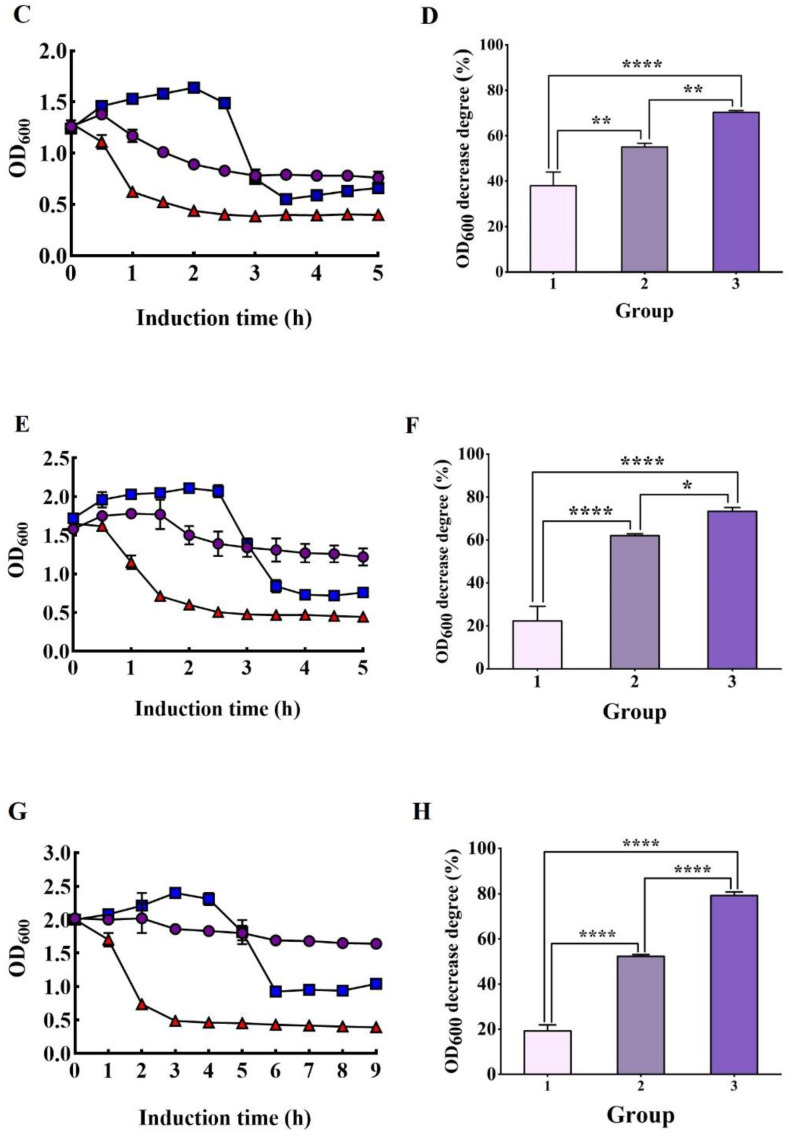
The lysis curves of *E. coli* BL21(DE3) harboring different lysis plasmids. The OD_600_ value of bacteria cells was measured to assess the lysis activity of bacteria (**A**,**C**,**E**,**G**). At the same time, the significance analysis of OD_600_ decrease degree of different lysis plasmids was conducted (**B**,**D**,**F**,**H**). The OD_600_ values at different times were expressed as mean ± standard deviation (S.D). The initial induction OD_600_ values were 0.8 (**A**,**B**), 1.2 (**C**,**D**), 1.6 (**E**,**F**), and 2.0 (**G**,**H**), respectively. Note: "ns" meant there was no significant difference. And “*”, “**”, “***” and “****” represented “*p* < 0.05”, “*p* < 0.01”, “*p* < 0.001”, and “*p* < 0.0001”, respectively, which had significant differences.

The most visual way to assess the difference in lysis activity between φX174-E and ID52-E was OD_600_ value decrease degree. Therefore, different initial induction OD_600_ values (low OD and high OD) were controlled to be the same before inducing the expression of lysis proteins E. When the initial induction OD_600_ value was low, there was no significant difference between lysis protein φX174-E and lysis protein ID52-E under the control of thermo-inducible lambda pL/pR-cI857 promoter, while there were significant differences between lysis protein ID52-E under the control of L-arabinose inducible promoter and the other two (Figure 1A,B). Furthermore, with the increase of initial induction OD_600_ value, the OD_600_ value of lysis protein ID52-E under the control of L-arabinose inducible promoter decreased the most, indicating that its lysis activity was the most optimal (Figure 1). As was shown in Table 2, contrary to the OD_600_ decrease degree, the lysis efficiency of lysis protein ID52-E under the control of L-arabinose inducible promoter was slightly lower than the other two, but there was no significant difference in the lysis efficiency among the three. Because the expression of lysis protein in bacteria harboring pBV220 plasmid was activated at 42 °C, while the high temperature affected bacterial growth, cell membrane composition and fluidity [33]. Moreover, as a membrane protein, the insertion of lysis protein into the cell membrane could reduce the cell growth rate and had a toxic effect on cell growth [34]. Therefore, it was speculated that the reason for this phenomenon was that the high temperature and the insertion of lysis protein on the cell membrane may cause a double burden on the bacteria, leading to a significant decrease in viable bacteria after induction.

And since the death of bacteria did’t mean the formation of BGs, the lysis efficiency can’t be used as the absolute standard of the lysis effect of lysis protein. As was shown in Table 2, the lysis efficiency of lysis protein ID52-E increased gradually with the increase of initial induction OD_600_ value. Although the initial induction OD_600_ value differed, the ultimate OD_600_ value after the lysis protein ID52-E expression dropped to similar levels. Therefore, the yield of BG was significantly increased.

**Figure 2:**
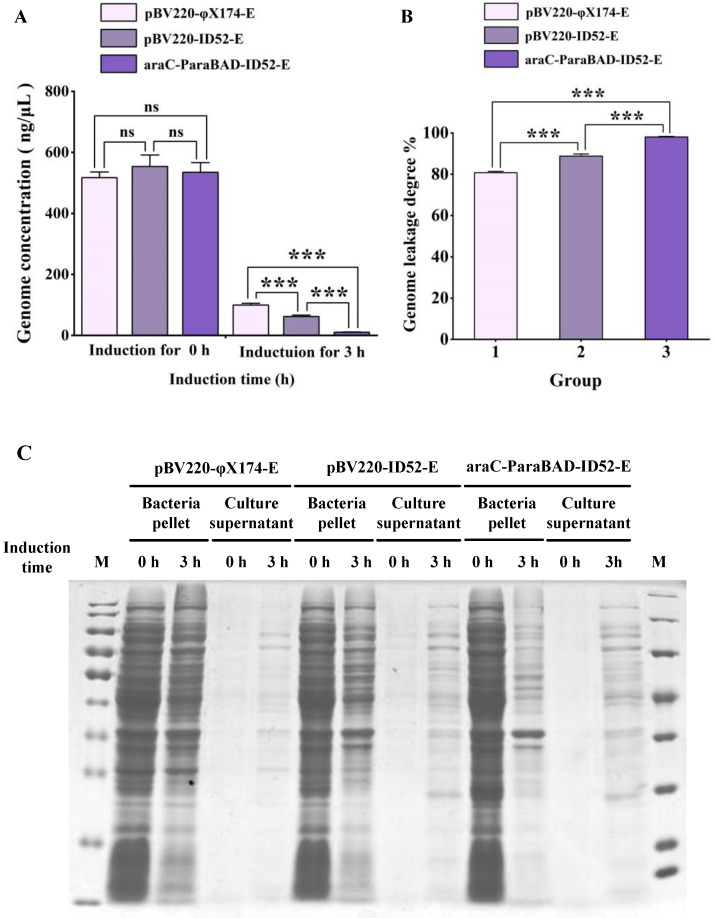
Characterization of bacterial genome and intracellular protein leakage. (**A**) Genomic concentrations of bacterial pellets before and after lysis, bacterial genome leakage (**B**) and intracellular protein leakage (**C**) in *E. coli* BL21(DE3) containing different plasmids. Note: “ns” represented "ns" meant there was no significant difference. And “***” represented “*p* < 0.001”, which had significant differences.

Consistent with the trend of lysis curve, the characterization results of bacterial genome leakage showed that *E. coli* BL21(DE3) containing araC-ParaBAD-ID52-E plasmid had the least residual amount of genome and the highest degree of genome leakage, while *E. coli* containing pBV220-φX174-E plasmid had the most residual amount of genome and the lowest degree of genome leakage. These results indicated that lysis protein ID52-E under the control of L-arabinose inducible promoter had the best lysis effect, which resulted in more genome flow to external media (Figure 2A,B). The results of SDS-PAGE also showed that *E. coli* BL21(DE3) containing araC-ParaBAD-ID52-E plasmid had the most intracellular protein leakage (Figure 2C).

### 3.3. Morphological Observation of E. coli BL21(DE3) BGs by SEM and TEM

**Figure 3:**
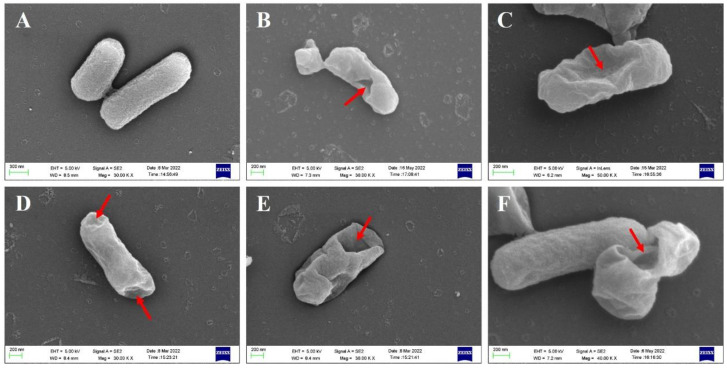

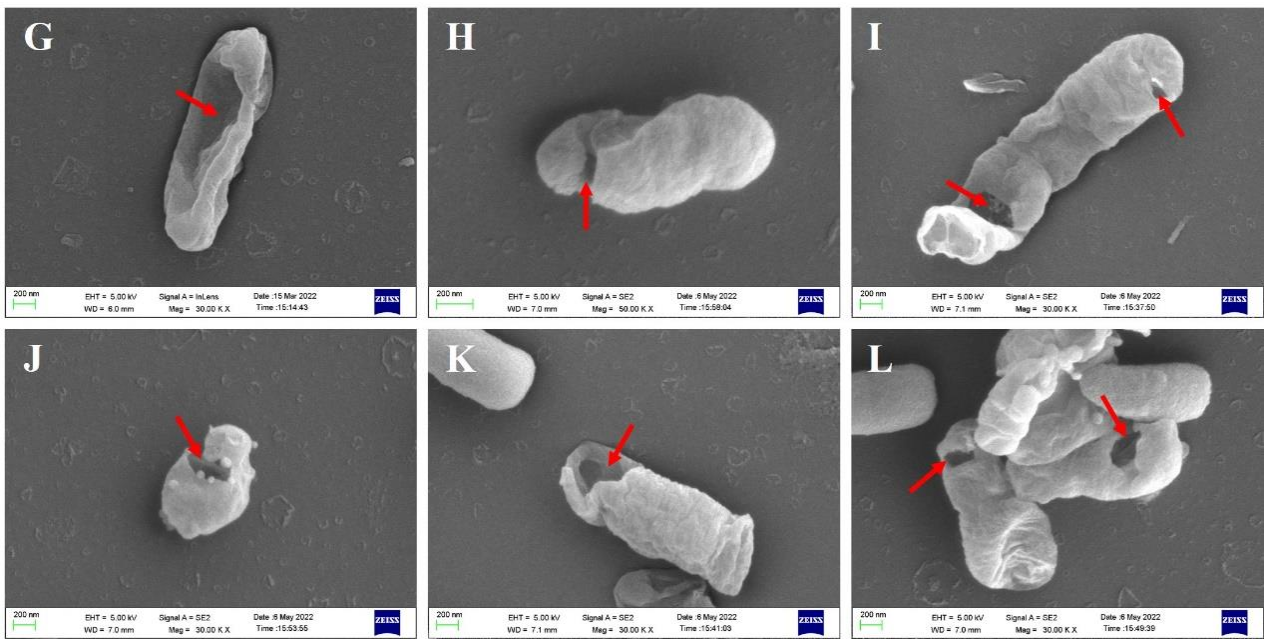
Morphological Observation of *E. coli* BL21(DE3) BGs by SEM. (**A**): Intact morphology of wild-type *E. coli* BL21(DE3); (**B**,**C**): *E. coli* BL21(DE3) ghost formed after lysis induced by plasmid pBV220-φX174-E; (**D**–**F**): *E. coli* BL21(DE3) ghost formed after lysis induced by plasmid pBV220-ID52-E; (**G**–**L**): *E. coli* BL21(DE3) ghost formed after lysis induced by plasmid araC-ParaBAD-ID52-E. The red arrow indicates the transmembrane tunnels.

BGs retained the natural surface morphological and structural characteristics of bacteria and could be observed by SEM, while effluent of bacterial contents could be identified by TEM. SEM images revealed that the overall morphology of BGs formed by lysis protein φX174-E and lysis protein ID52-E was not much different from that of intact bacterial cells, and both retained the same membrane structure as that of natural living bacteria (Figure 3). At the beginning of lysis, the cell membrane at the lysis pore shrinked into the lumen of the cell, but the cell membrane may collapse inward when the cell contents were all expelled. Similar to the reported lysis protein φX174-E [19,20], transmembrane channels of ID52-E were not randomly distributed on the envelope, but mainly formed at the equatorial center, poles or position near the poles of the bacteria. Moreover, except for a few BGs that formed two transmembrane channels, most of BGs formed only one pore. From SEM images, it was observed that the transmembrane lysis channels formed by ID52-E was larger than those formed by φX174-E, which might be the reason why the lysis activity of ID52-E was stronger than φX174-E.

**Figure 4:**
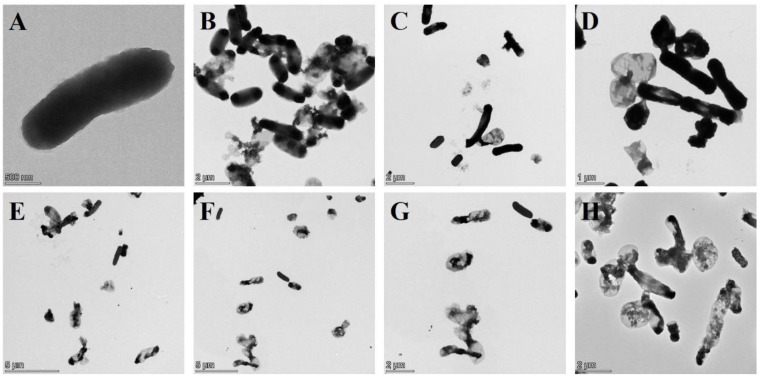

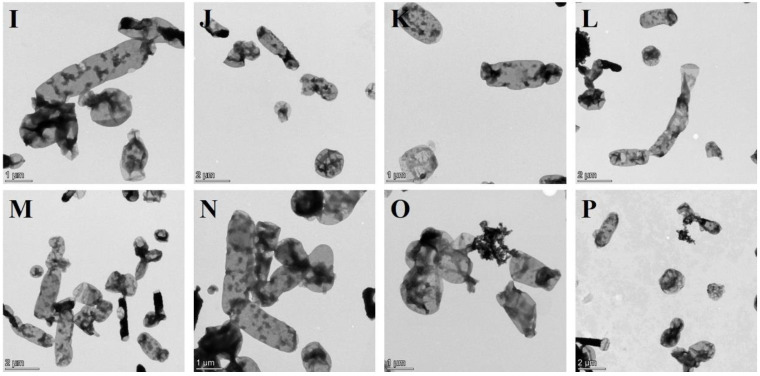
Morphological Observation of *E. coli* BL21(DE3) BGs by TEM. (**A**): Intact morphology of wild-type *E. coli* BL21(DE3); (**B**–**D**): *E. coli* BL21(DE3) ghost formed after lysis induced by plasmid pBV220-φX174-E; (**E**–**H**): *E. coli* BL21(DE3) ghost formed after lysis induced by plasmid pBV220-ID52-E; (**I**–**P**): *E. coli* BL21(DE3) ghost formed after lysis induced by plasmid araC-ParaBAD-ID52-E.

TEM images manifested that the untreated wild-type *E. coli* BL21(DE3) showed uniform dark color due to containing DNA, protein and other cell contents (Figure 4A), while the color of the lumen of *E. coli* BL21 ghosts were relatively light due to the devoid of intracellular cytoplasm (Figure 4B–P). As shown in Figure 4B–D, BGs formation rate was the lowest after the lysis induced by plasmid pBV220-φX174-E where most of the bacteria had not excreted cell cytoplasm. However, BGs formation rate was the highest after lysis induced by plasmid araC-ParaBAD-ID52-E where bacteria basically expelled cytoplasm to form BGs, except for a few bacteria (Figure 4I–P). TEM results were consistent with the characterization results of DNA leakage and protein leakage in Figure 2.

### 3.4. Detecting the Expression Level and Expression Location of Lysis Protein E by Western Blot

**Figure 5:**
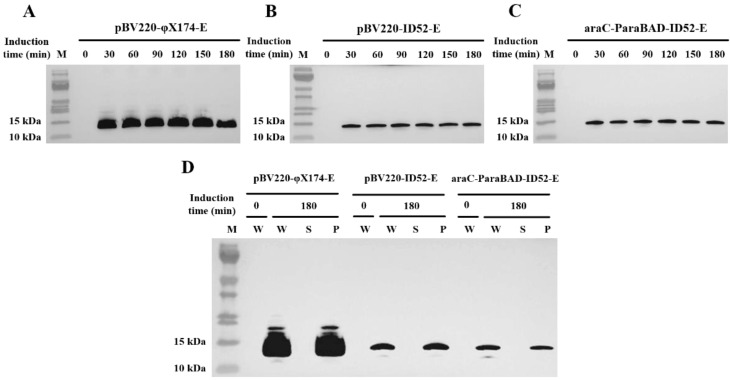
The expression levels and expression location of lysis protein E at different induction times were detected by Western blot. (**A**–**C**): The whole lysis solution of *E. coli* BL21(DE3) harboring pBV220-φX174-E, pBV220-ID52-E and araC-ParaBAD-ID52-E plasmid, respectively; (**D**) The expression location of lysis protein E. M: 26616 prestained protein marker, W: whole lysis solution; S: supernatant; P: precipitate.

Western blot of the samples manifested that the expression levels of different lysis protein E were significantly different in *E. coli* BL21(DE3) containing different plasmid (Figure 5A–C). The expression levels of lysis protein in *E. coli* BL21(DE3) containing pBV220-φX174-E was significantly higher than *E. coli* BL21(DE3) containing pBV220-ID52-E or araC-ParaBAD-ID52-E plasmid, while the expression levels of different plasmids containing lysis protein ID52-E were almost the same. It was speculated that the molecular weight of the lysis protein φX174-E (11.64 kDa) was lower than that of ID52-E (12.77 kDa), so the expression quantity of lysis protein φX174-E was higher in the same induction time. Further, there is possible that the amount of protein required by lysis protein ID52-E to lyse host bacteria was far less than that required by lysis protein φX174-E to lyse host bacteria. When the number of lysis protein ID52-E synthesis reached the threshold of lysis host bacteria, lysis pores formed on the cell membrane, and the host bacteria began to lyse and die, and the lysis protein ID52-E synthesis was terminated, resulting that the expression of ID52-E was lower than φX174-E. And contrary to the expression levels of the two lysis proteins E, the lysis activity of protein ID52-E with lower expression level was significantly higher than those of φX174-E with higher expression level, indicating that the lysis activity of protein ID52-E was so stronger that lysis bacteria required less protein ID52-E.

**Figure 6:**
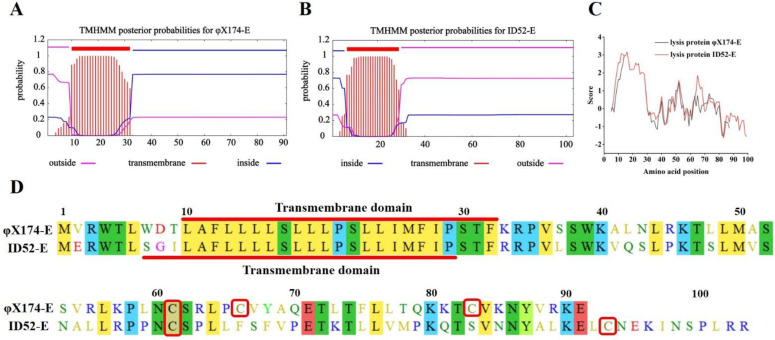
Transmembrane domain prediction, hydrophilicity analysis and amino acids sequence alignment of lysis proteins E. (**A**,**B**): Transmembrane domain prediction of proteins φX174−E and ID52−E performed by TMHMM Server 2.0, respectively; (**C**): The hydrophilicity analysis of proteins φX174−E and ID52−E performed by ExPASy online tool Protscale; (**D**): The amino acids sequence alignment of lysis protein performed by Molecular Evolutionary Genetics. The NCBI Reference Sequence of lysis protein φX174−E: NP_040709.1; The NCBI Reference Sequence of lysis protein ID52−E: YP_512455.1. The color represented the similarity of amino acids, and the same color meant that the amino acid residues of the two lysis proteins were the same or the characteristics were very similar. The red box represented the cysteine contained in the lysis proteins.

In order to understand the possible causes of the difference in the lysis activity of the two lysis proteins, sequence alignment of amino acids of lysis protein was performed by Molecular Evolutionary Genetics. At the same time, TMHMM Server 2.0 was used to predict and analyze the transmembrane region of lysis protein, and ExPASy online tool Protscale was used to further analyze the hydrophilicity and hydrophobicity of lysis protein. It was found that the transmembrane domain of the lysis protein φX174-E was the 10~32 amino acids, and that of the lysis protein ID52-E was the 7~29 amino acids (Figure 6A–C). The length and location of the transmembrane domain of the two proteins were similar, and the 21th amino acid was proline, indicating that the transmembrane domain was very conserved in lysis protein of *Microviridae* (Figure 6A,B,D), while the hydrophilicity of the C-terminal of the two lysis proteins was significantly different (Figure 6C). Previous experiments [35,36,37] showed that transmembrane domain was a necessary condition for the lysis activity of the protein φX174-E, and the C-terminal affected the lysis effect of the protein, significantly. In addition, the C-terminal of φX174-E has three cysteines, while the C-terminal of ID52-E has only two cysteines. As is known to all, cysteine is the prerequisite for the formation of disulfide bonds, and previous studies have shown that the lysis protein φ174-E played a role as a polymer [38]. Because more cysteine may mean that the lysis protein may have a more complex structure. So it was reasonable to speculate that this may be one of the reasons for the significant difference in the lysis activity of the two proteins. In general, C-terminal may play an important role in the lysis activity of lysis proteins, in which the hydrophilicity and hydrophobicity of C-terminal and cysteine content may play a major role.

According to the report, the lysis protein φX174-E is a membrane protein and should be detected in the precipitate after centrifugation [39]. In accordance with the report, Western blot results revealed that both the protein φX174-E and protein ID52-E could be detected in the whole solution and precipitation, but not in the supernatant, suggesting that both lysis protein φX174-E and lysis protein ID52-E were expressed on the membranes (Figure 5D).

### 3.5. Construction of Engineered Strain EcN with araBAD Deletion Mutation and ST with araBAD Deletion Mutation

In order to test the application scope of the recombinant expression of protein ID52-E under the control of L-arabinose inducible promoter to prepare BGs, the system was applied to EcN and ST. However, different from *E. coli* BL21(DE3), EcN and ST have a faster consumption rate of inducer L-arabinose, so it was easy to cause insufficient supply of inducer and thus reduced the lysis rate. At the same time, a certain amount of L-arabinose can promote the growth of EcN and ST, and finally leaded to the phenomenon that the growth rate was faster than the lysis rate and the lysis curve OD_600_ value rised, resulting in a diminished output of BGs. Therefore, in order to reduce the use of inducers and increase the yield of BG, it was necessary to knock out *araBAD* gene related to L-arabinose consumption. And the λ Red homologous recombination system was used for gene knockout.

**Figure 7:**
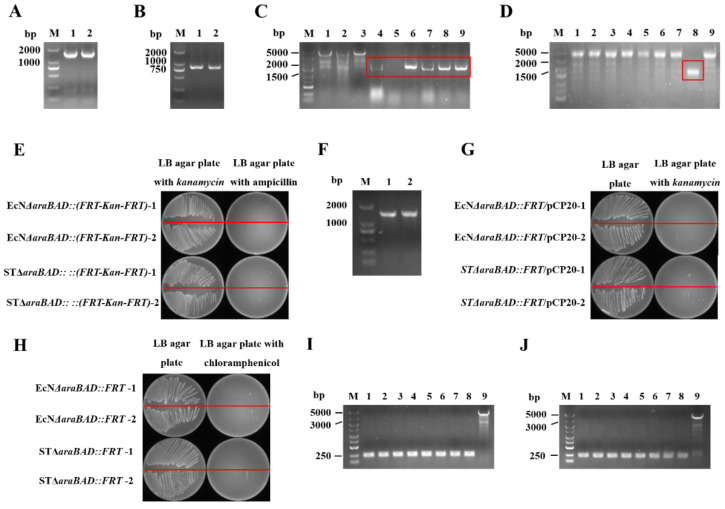
Construction of engineering strain EcN with *araBAD* deletion mutation and ST with *araBAD* deletion mutation. (**A**) The amplification of linear FRT-kanamycin-FRT fragment, band 1: linear FRT-kanamycin-FRT fragment containing 56-nt homology arms targeting the *araBAD* gene of EcN, band 2: linear FRT-kanamycin-FRT fragment containing 56-nt homology arms targeting the *araBAD* gene of ST; (**B**) The electrotransformation of pKD46, band 1: EcN clone, band 2: ST clone; (**C**,**D**) Colony PCR to identify the deletion of *araBAD* gene and the insertion of *kanamycin* gene of EcN or ST, and positive bands are marked in a red box; (**E**): The loss of pKD46 plasmid; (**F**): The electrotransformation of pCP20, band 1: EcN*ΔaraBAD::kanamycin* strain, band 2: ST *ΔaraBAD::kanamycin* strain; (**G**): Excision of *kanamycin* gene; (**H**): The loss of pCP20 plasmid; (**I**,**J**): Colony PCR to identify the deletion of *araBAD* gene engineered strain of EcN or ST, bands 1-8: engineered strain of EcN or ST, band 9: EcN wild type or ST wild type.

The linear FRT-kanamycin-FRT fragment of 1477 bp containing 56-nt homology arms targeting the *araBAD* gene was amplified and then was identified by 1% agar electrophoresis analysis (Figure 7A). The detection of 792 bp bands showed that pKD46 plasmids were successfully transited into EcN and ST (Figure 7B). And a band of approximately 1600 bp were visualized in the strain with the deletion of *araBAD* gene and the insertion of kanamycin, while bands of approximately 4200 bp were detected in the wild-type (Figure 7C,D). Clones that were able to grow on LB agar plates containing kanamycin but not LB agar plates containing ampicillin proved successful in losing the pKD6 plasmid (Figure 7E). Next, the pCP20 plasmid was successfully transferred into the ECN*ΔaraBAD::kanamycin* and ST*ΔaraBAD::kanamycin*, which were verified by the detection of a 1387 bp band (Figure 7F). After induction at 42 °C, clones sensitive to kanamycin were screened, which proved that kanamycin fragment was successfully excised (Figure 7G). As with the loss of pKD46 plasmid, the pCP20 plasmid was lost by culturing kanamycin-sensitive clones at 42 °C, which could be verified by measuring the sensitivity to chloramphenicol (Figure 7H). Eventually, bands of approximately 250 bp were visualized in the engineered strains EcN*ΔaraBAD::FRT* and ST*ΔaraBAD::FRT*, while bands of approximately 4200 bp were detected in wild-type, indicating that the *araBAD* gene was deleted from wild-type strains (Figure 7I,J). For further verification, PCR products were sent for sequencing (data not shown).

### 3.6. Growth, Lysis, and Characterization of EcN BGs and ST BGs

**Figure 8:**
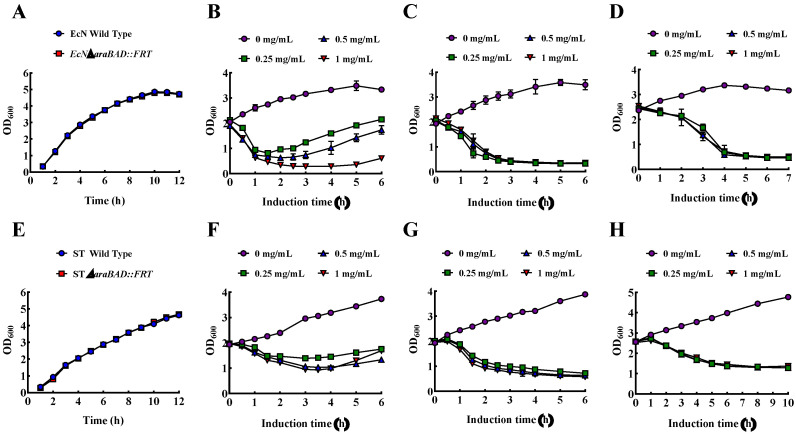
The growth curves and lysis curves of *E. coli* Nissle 1917*ΔaraBAD::FRT* containing plasmid araC-ParaBAD-ID52-E and ST*ΔaraBAD::FRT* containing plasmid araC-ParaBAD-ID52-E engineering strain. (**A**): The growth curves of EcN WT and EcN*ΔaraBAD::FRT*; (**B**) The lysis curves of EcN WT when the initial OD_600_ reached 2.0; (**C**) The lysis curves of EcN*ΔaraBAD::FRT* when the initial OD_600_ reach 2.0; (**D**) The lysis curves of EcN*ΔaraBAD::FRT* when the initial OD_600_ reached 2.5; (**E**): The growth curves of ST WT and ST*ΔaraBAD::FRT*; (**F**) The lysis curves of ST WT when the initial OD_600_ reached 2.0; (**G**) The lysis curves of ST*ΔaraBAD::FRT* when the initial OD_600_ reached 2.0; (**H**) The lysis curves of ST*ΔaraBAD::FRT* when the initial OD_600_ reached 2.5.

There was no significant difference between the growth rate of EcN wild-type and EcN engineering strain, and the growth rate of ST wild-type and ST engineering strain, indicating that *araBAD* gene knockout had no significant effect on the growth of EcN and ST strain in LB broth (Figure 8A,E). When the initial OD_600_ was about 2.0, under the induction of different L-arabinose concentration, the wild type *E. coli* Nissle 1917 decreased to a certain OD_600_ and began to rise (Figure 8B). However, the concentration of L-arabinose had no effect on the lysis of *E. coli* Nissle 1917*ΔaraBAD::FRT*, and the lowest OD_600_ value was about 0.3 (Figure 8C), indicating that the knockout of L-arabinose consumption related genes *araBAD* effectively reduced the consumption of inducer L-arabinose in *E.coli* Nissle 1917. What’s more, effective lysis can still occur when the initial induction was up to 2.5 (Figure 8D). The lysis of ST*ΔaraBAD::FRT* engineering strain was similar to that of *E.coli* Nissle 1917*ΔaraBAD::FRT* (Figure 8F–H). The efficient and high yield preparation of EcN BGs and ST BGs demonstrated the feasibility of recombinant expression of *E. coli* phage ID52-E under the control of L-arabinose inducible promoter, significantly improving the yield of BGs.

**Figure 9:**
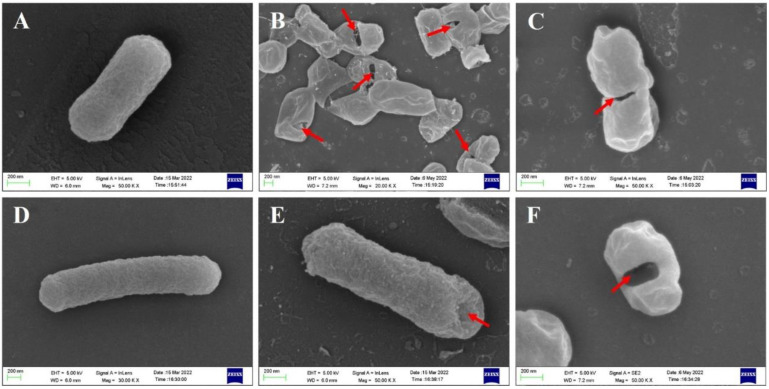
Morphological observation and cellular contents observation of EcN BGs and ST BGs by SEM. (**A**): Untreated control EcN cells; (**B**,**C**): EcN ghosts; (**D**) untreated control ST cells; (**E**,**F**): ST ghosts. The red arrows indicated the lysis transmembrane tunnels.

**Figure 10:**
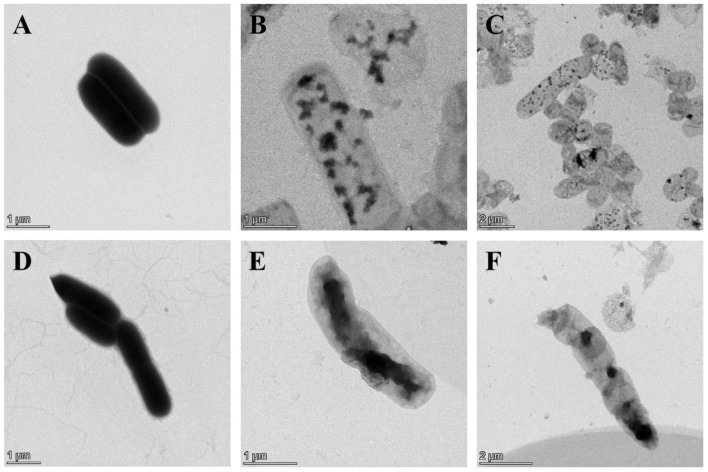
Morphological observation and cellular contents observation of EcN BGs and ST BGs by TEM. (**A**): untreated control EcN cells; (**B**,**C**): EcN ghosts; (**D**) untreated control ST cells; (**E**,**F**): ST ghosts.

As were shown in Figure 9, the EcN BGs and ST BGs maintained their intact cell morphology and membrane structures whose transmembrane tunnels were located at the equator or poles of bacteria cells (Figure 9B,C,E,F). In comparison with the untreated EcN and ST, the discharge of cellular contents of the EcN BGs and ST BGs were visualized by TEM (Figure 10B,C,E,F).

## 4. Discussion

BGs referred to the nonliving empty bacterial shell without cytoplasmic contents, but retained original cell morphology and the same antigenicity of living counterparts, which could evoke potent humoral immunity response, cellular immunity response and mucosal immunity response [1,2]. As potential vaccine candidates, BGs had inherent adjuvant properties without adding extra adjuvant, while commercial inactivated vaccines were weak in immunogenicity and required additional adjuvants to enhance the immune response [40]. Compared with live attenuated vaccines, due to the lack of cytoplasm, BGs did’t have the possibility of reverting to strong virulence and horizontal genetic recombination [40]. Further, BGs conferred an efficient delivery vehicle that enhance immunogenicity of protein subunits vaccines and DNA-encoded antigens [18]. Moreover, not only can the empty cavity of BGs be loaded with drug [41], DNA [42] and pesticide [43], but also can the protein antigens be expressed at the inner membrane, outer membrane [10] and periplasmic space [44] of BGs before lysis. The good qualities of BGs made them have a broad and good application prospect in immunology and drug delivery.

The vaccine control effect and drug delivery performance of BGs have been studied, which showed that BGs were excellent vaccine candidates and delivery systems. However, no BGs has been officially approved and commercialized. One of the reasons was that the production of BGs was very low. In recent years, how to effectively improve the production of BGs has been perplexing many scholars, and some scholars have conducted researches on this [22,23,24], but all of them focused on the lysis protein E of *E. coli* phage φX174. However, whether other phage lysis proteins can be applied to the preparation of BGs has not been reported. 

Like *E. coli* phage φX174, *E. coli* phage ID52 belongs to the *Microviridae* family with a ubiquitous small isometric ssDNA genome, which can infect numerous bacterial species in different bacterial phyla [30,45]. Contrary to the multi-gene lysis system of double-strand nucleic acid phages, which need at least one muralytic enzyme, the endolysin, and one cytoplasmic membrane protein, the holin, that actively attack the integrality of the membrane at a genetically determined time, lysis systems of phages in the *Microviridae* family only require encode a single lysis protein, which does not encode degradation activity but induces host cells to undergo autolysis [46,47]. Thus, the lysis of *Microviridae* family is not precisely scheduled, but occurs at a time dependent on the cell cycle and/or the phase of host growth [46].

Previous studies have shown that the lysis activity of protein φX174-E was affected by the growth stage of host bacteria. Only when the host bacteria were in the logarithmic phase, can protein φX174-E effectively lyse host bacteria [24], resulting the low yield of BGs. In order to improve the yield of BGs, the lysis protein of *E. coli* phage ID52 were introduced into the production of BGs to investigate whether the lysis activity of protein ID52-E was also affected by the growth phase of host bacteria. The results showed that the lysis effect of protein φX174-E was gradually decreased with the increase of initial induction OD_600_, while the lysis effect of protein ID52-E was not significantly affected, indicating that the growth stage of host bacteria had no significant effect on the lysis of protein ID52-E. In contrast to the lysis activity of the protein φX174-E and ID52-E, Western blot analysis revealed that the expression level of protein φX174-E was significantly higher than ID52-E, indicating that the protein φX174-E required for the lysis of same number bacteria was significantly higher than that required by ID52-E. The results of amino acid sequence alignment, transmembrane domain prediction and hydrophilicity prediction showed that the N-terminal and transmembrane domain sequences of protein φX174-E and ID52-E were similar, but the length, hydrophilicity and cysteine content of C-terminal were different, suggesting that the difference in lysis activity of the two proteins was mainly attributed to the C-terminal which required more experimental verifications to confirm in spite of that it has been confirmed that the C-terminal has a great influence on the lysis of protein φX174-E [35,36,37]. As it was reported, protein φX174-E formed transmembrane channel in the form of polymer [38], so it was speculated that the number of cysteines at C-terminal played an important role by affecting the conformation of the polymer.

Studies have shown that the lysis activity of the protein φX174-E depends on the lipid composition and fluidity of the cell membranes [48]. The results showed that the lysis activity of protein ID52-E under the control of L-arabinose-inducible promoter were significantly higher than that of ID52-E under the control of temperature-inducible promoter despite almost the same amount of protein being expressed, which was speculated that high temperature affected the cell growth, cell membrane composition and fluidity [33], thus affecting the membrane insertion of lysis protein ID52-E. The successful preparation of EcN BGs and ST BGs confirmed the feasibility of L-arabinose-inducible ID52-E lysis system, and it was speculated that the lysis system can also be applied to the preparation of other *Enterobacteriaceae* BGs. Recently, a novel and efficient high-yield method for preparing BGs by the mutant of lysis protein φX174-E under the control of T7 promoter and plasmid pLysS were constructed, whose initial induction OD_600_ also can reached 2.5 [24]. However, the T7 promoter needed to be induced by IPTG, which was not only costly, but also had a certain toxic effect [49]. On the contrary, the inducer L-arabinose had the advantages of low cost and edible, so the preparation method of BGs in this paper is safer and cheaper.

However, although some achievements have been obtained in this study, there were still some shortcomings to be improved. Whether the lysis system of lysis protein ID52-E under the control of L-arabinose inducible promoter could be applied to the efficient preparation of other host bacterial ghosts remains to be further studied. On the one hand, it is necessary to consider whether the protein ID52-E can lyse host bacteria, and on the other hand, it is necessary to consider whether the host bacteria can initiate the L-arabinose inducible promoter, although the lysis plasmid araC-ParaBAD-ID52-E contains regulatory elements regulating the L-arabinose inducible promoter. In addition, whether the L-arabinose consumption related gene *araBAD* needs to be knocked out can be determined according to the rate of L-arabinose consumption by host bacteria.

And it has been reported that protein φX174-E mediated cell lysis mechanism was related to cell division, and its kinetics was species-specific, which was speculated to be the same for protein ID52-E [21,24]. Therefore, the maximum initial lysing OD_600_ and minimum lysing OD_600_ of different host bacteria were also different, which needed to be optimized according to different host bacteria. Further, the recombinant expression of lysis protein ID52-E was used to successfully prepare *E. coli* BL21(DE3) ghosts, EcN ghosts and ST ghosts with high efficiency and high yield, but the immune effect was not performed, which still needs to be further expanded. And the lysis efficiency of protein ID52-E under control of L-arabinose inducible promoter was lower than that of protein φX174-E under control of temperature-inducible promoter, but the death of host bacteria didn’t represent the formation of BGs. As observed by TEM (Figure 4), protein φX174-E under control of temperature-inducible promoter has a high lysis efficiency (Table 2), but most of the cell inclusions of host bacteria have not been discharged, manifesting the BGs formation rate was low, while protein ID52-E under control of L-arabinose inducible promoter, whose BGs formation rate was high because the inclusions of host bacteria in the majority have been discharged, has a slightly lower lysis efficiency (Table 2). Therefore, it was necessary to find other methods for quantitative detection of BGs formation rate.

Finally, inspired by other scholars’ research on targeted tumor therapy with EcN BGs [50], because EcN live bacteria can colonize and replicate in tumors, it was speculated that it was possible that some anticancer factors were expressed in EcN, which colonize directed to the tumors, and then L-arabinose, a five carbons sugar [51], was administered to induce EcN lysis to release drugs, so as to achieve the purpose of targeted tumor therapy.

## 5. Conclusions

In summary, this study not only described a novel and excellent lysis protein ID52-E for BGs preparation for the first time, but established an efficient high-yield BGs preparation system with the recombinant expression of *E. coli* phage ID52-E under the control of L-arabinose inducible promoter, which provided the possibility for the efficient preparation of *Enterobacteriaceae* BGs and significantly improved the yield of BGs, laying a technical foundation for the large-scale production and application of BGs.

## Figures and Tables

**Table 1:** Bacterial strains, plasmids and primers used in this research.

Strains Plasmids Primers	Description	Source
**Strains**		
DH5α	Host cells for plasmid amplification	TIANGEN BIOTECH, Beijing, China
BL21(DE3)	Host cells for protein expression	TIANGEN BIOTECH, Beijing, China
*E. coli* Nissle 1917	Wild type	Mutaflor, Germany
*E. coli* Nissle 1917 Δ*araBAD::FRT*	Deletion of *araBAD* gene and insertion of *FRT* locusin *E. coli* Nissle 1917	This study
ST	Wild type of S*almonella enterica* subsp*. enterica* serovar*Typhimurium str.* ATCC 14028	Institute of Microbiology, Guangdong Academy of Science, China
ST Δ*araBAD::FRT*	Deletion of *araBAD* gene and insertion of *FRT* locus in ST	This study
**Plasmids**		
pBV220-sGFP-Chl	The template of constructing expression vector of lysis gene E	Our lab
pET29a-φX174-E	The template of constructing expression vector ofgene *φX174-E*	Our lab
pET29a-ID52-E	The template of constructing expression vector of gene *ID52-E*	Our lab
pBV220-φX174-E	Lysis plasmid used in this study	This study
pBV220-ID52-E	Lysis plasmid used in this study	This study
araC-ParaBAD-ID52-E	Lysis plasmid used in this study	This study
pKD4	Plasmid for λ Red homologous recombination	Our lab
pKD46	Plasmid for λ Red homologous recombination	Our lab
pCP20	Plasmid for λ Red homologous recombination	Our lab
**Primers**		
φX174-E-F	TTGGTTAAAAATTAAGGAGGAATTCATGGTACGCTGGACTTTGTG	
φX174-E-R	ACAGCCAAGCTTGGCTGCAGTTATTTTTCAAACTGCGGATG	
ID52-E-F	TAAAAATTAAGGAGGAATTCATGGAACGCTGGACCTTAAG	
ID52-E-R	ACAGCCAAGCTTGGCTGCAGTTATTTTTCAAACTGCGGATG	
araC-ParaBAD-F	TGCGCCGACCAGAACACCTTGCCGATTATGACAACTTGACGGCTACA	
araC-ParaBAD-R	TGCCGCTTAAGGTCCAGCGTTCCATTTTTTATAACCTCCTTAGAGCTCG	
linearized pBV220-ID52-E-F	ATGGAACGCTGGACCTTAAG	
linearized pBV220-ID52-E-R	TCGGCAAGGTGTTCTGGT	
P1	ATGACACCGGACATTATCCTG	
P2	GTGCTTTCAGTGGATTTCGG	
P3	TTTTTCGCAACTCTCTACTGTTTCTCCATACCCGTTTTTTTGGATGGAGTGAAACGGTGTAGGCTGGAGCTGCTTC	
P4	CTGGTTTCGTTCCAAAACCAAAATTTATTTTGATTGGCTGTGGTTTTATACAGTCACATATGAATATCCTCCTTAG	
P5	TTTGCCGCGACTCTCTACTGTTTCTCCATACCTGTTTTTCTGGATGGAGTAAGACGGTGTAGGCTGGAGCTGCTTC	
P6	TATATCACCGACCAGATTCATCAACGCGCCCCCCATGGGAGCGTTTTTAGAGGCACATATGAATATCCTCCTTAG	
P7	TTAGCGGATCCAGCCTGA	
P8	TGCAGCATTCGCAGATCG	
P9	GATTAGCGGATCCTGCCTGA	
P10	TATCAAAGCGCATTTGCTGAA	
P11	AAGGGATAAATATCTAACACCGTGC	
P12	ACGGCATAGTGCGTGTTTATG	

Red letters indicate the 50-nt homology arms targeting the *araBAD* gene.

**Table 2:** The lysis efficiency of *E. coli* BL21(DE3) harboring different lysis plasmids.

Initial Induction OD_600_ Values	Lysis Efficiency (%)		
	pBV220-φX174-E	pBV220-ID52-E	araC-ParaBAD- ID52-E
0.8	100	99.984	99.755
1.2	100	99.994	99.994
1.6	100	99.998	99.997
2.0	100	100	99.998

Note: the data were presented in the format of mean values.

## Data Availability

Not Applicable.

## Supplementary Materials

The following supporting information can be downloaded at: https://www.mdpi.com/article/10.3390/bioengineering9070300/s1, Figure S1: The construction of lysis protein E expressing Plasmid pBV220-φX174-E (A), pBV220-ID52-E (B) and araC-ParaBAD-ID52-E (C), Figure S2: Agarose gel electrophoresis analysis of colony PCR of *E. coli* DH5α colonies containing plasmids pBV220-φX174-E (A, lane 1-2), pBV220-ID52-E (A, lane 3-4) and araC-ParaBAD-ID52-E (B), respectively.
