# Supplementary material for: Efficient Robust Yield Method for Preparing Bacterial Ghosts by Escherichia coli Phage ID52 Lysis Protein E
Source: Bioengineering (Basel). 2022 Jul 7;9(7):300. doi: 10.3390/bioengineering9070300 (PMC9311611; doi:10.3390/bioengineering9070300)
Supplement: Supplementary file 1 [file bioengineering-09-00300-s001.zip › bioengineering-1715936-supplementary.pdf]

bioengineering

MDPI

Supplementary Materials

Efficient Robust Yield Method for Preparing Bacterial Ghosts by *Escherichia coli* Phage ID52 Lysis Protein E

Yi Ma [1,2,*], Wenjun Zhu [1], Guanshu Zhu [1], Yue Xu [1], Shuyu Li [1], Rui Chen [3], Lidan Chen [4] and Jufang Wang [1,*]

[1] School of Biology and Biological Engineering, South China University of Technology, Guangzhou 510006, China; 201920146370@mail.scut.edu.cn (W.Z.); 202021049689@mail.scut.edu.cn (G.Z.); 202121050169@mail.scut.edu.cn (Y.X.); 201936500491@mail.scut.edu.cn (S.L.)
[2] Guangdong Provincial Key Laboratory of Fermentation and Enzyme Engineering, South China University of Technology, Guangzhou 510006, China
[3] Bionavi Life Sciences Co., Ltd., Shenzhen 518118, China; chenruicr21@163.com
[4] Department of Laboratory Medicine, General Hospital of Southern Theater Command of PLA, Guangzhou 510010, China; clidangz@163.com
* Correspondence: bimayikobe@scut.edu.cn (Y.M.); jufwang@scut.edu.cn (J.W.)

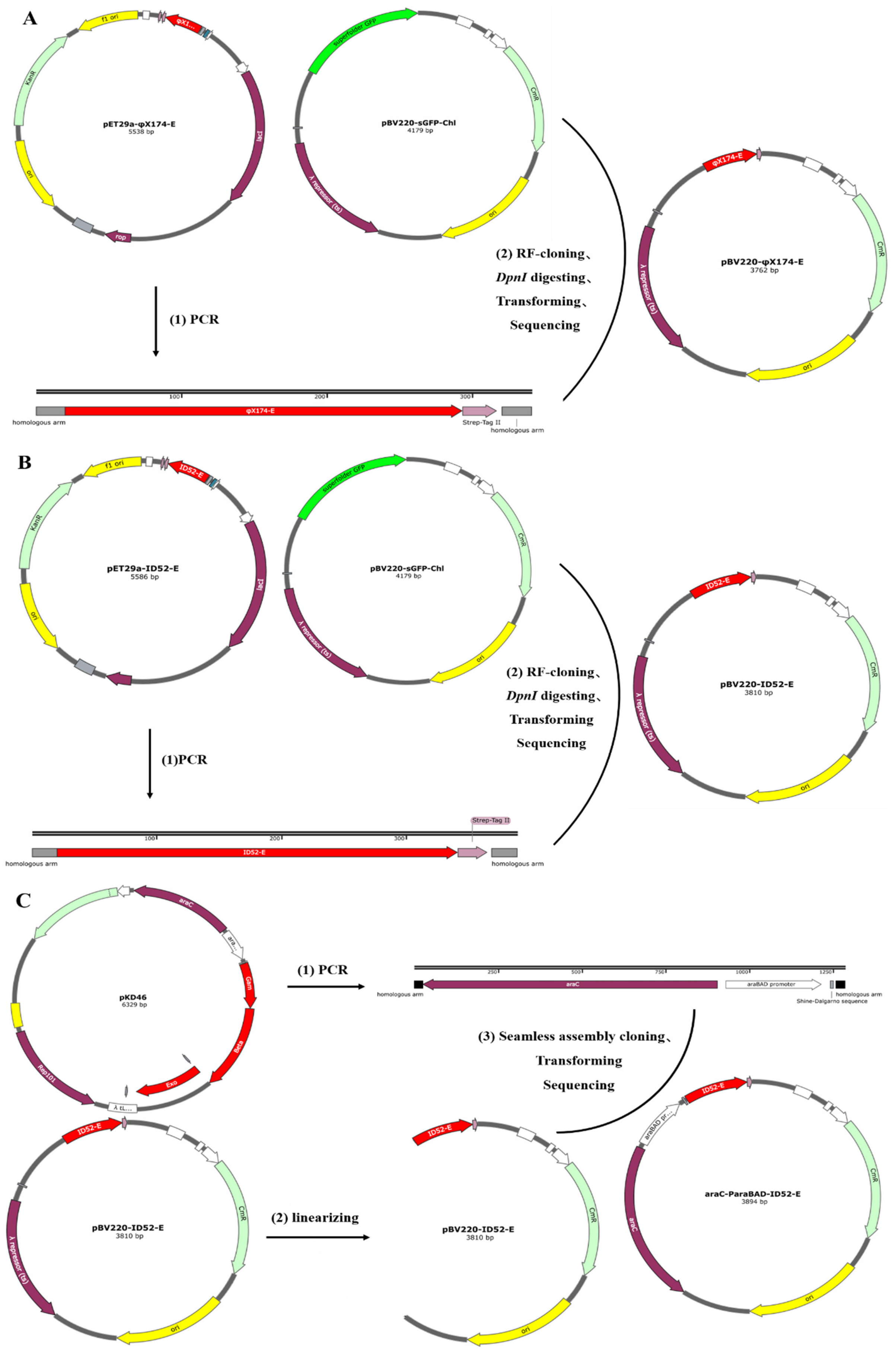
A
f1 ori
KanR
ori
lacI
rop
pET29a-φX174-E
5538 bp
superfolder GFP
CmR
λ repressor (ts)
ori
pBV220-sGFP-Chl
4179 bp
(1) PCR
(2) RF-cloning、
DpnI digesting、
Transforming、
Sequencing
φX174-E
pBV220-φX174-E
3762 bp
homologous arm
φX174-E
Strep-Tag II
homologous arm
B
pET29a-ID52-E
5586 bp
ID52-E
pBV220-sGFP-Chl
4179 bp
(1)PCR
(2) RF-cloning、
DpnI digesting、
Transforming
Sequencing
pBV220-ID52-E
3810 bp
Strep-Tag II
ID52-E
homologous arm
homologous arm
C
araC
Gam
Beta
Exo
Rep101
pKD46
6329 bp
(1) PCR
araC
araBAD promoter
homologous arm
homologous arm
Shine-Dalgarno sequence
(3) Seamless assembly cloning、
Transforming
Sequencing
pBV220-ID52-E
3810 bp
(2) linearizing
pBV220-ID52-E
3810 bp
araC-ParaBAD-ID52-E
3894 bp

Figure S1. The construction of lysis protein E expressing Plasmid pBV220-φX174-E (**A**), pBV220-ID52-E (**B**) and araC-ParaBAD-ID52-E (**C**).

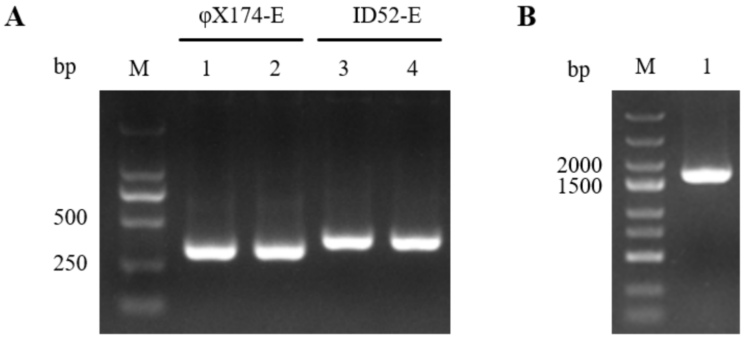

Figure S2. Agarose gel electrophoresis analysis of colony PCR of *E. coli* DH5α colonies containing plasmids pBV220-φX174-E (**A**, lane 1 – 2), pBV220-ID52-E (**A**, lane 3 – 4) and araC-ParaBAD-ID52-E (**B**), respectively.
